# Structural and Functional Consequences of Connexin 36 (Cx36) Interaction with Calmodulin

**DOI:** 10.3389/fnmol.2016.00120

**Published:** 2016-11-18

**Authors:** Ryan C. F. Siu, Ekaterina Smirnova, Cherie A. Brown, Christiane Zoidl, David C. Spray, Logan W. Donaldson, Georg Zoidl

**Affiliations:** ^1^Biology Program, York University, TorontoON, Canada; ^2^Chemistry Program, York University, TorontoON, Canada; ^3^Psychology Program, York University, TorontoON, Canada; ^4^Dominick P. Purpura Department of Neuroscience, Albert Einstein College of Medicine, New YorkNY, USA

**Keywords:** electrical synapse, connexins, calmodulin, CaMKII, plasticity, protein interaction

## Abstract

Functional plasticity of neuronal gap junctions involves the interaction of the neuronal connexin36 with calcium/calmodulin-dependent kinase II (CaMKII). The important relationship between Cx36 and CaMKII must also be considered in the context of another protein partner, Ca^2+^ loaded calmodulin, binding an overlapping site in the carboxy-terminus of Cx36. We demonstrate that CaM and CaMKII binding to Cx36 is calcium-dependent, with Cx36 able to engage with CaM outside of the gap junction plaque. Furthermore, Ca^2+^ loaded calmodulin activates Cx36 channels, which is different to other connexins. The NMR solution structure demonstrates that CaM binds Cx36 in its characteristic compact state with major hydrophobic contributions arising from W277 at anchor position 1 and V284 at position 8 of Cx36. Our results establish Cx36 as a hub binding Ca^2+^ loaded CaM and they identify this interaction as a critical step with implications for functions preceding the initiation of CaMKII mediated plasticity at electrical synapses.

## Introduction

Electrical and chemical synapses can form mixed synapses conferring synchronous activity among coupled neurons in the vertebrate nervous system (Hamzei-Sichani et al., 2012; Nagy et al., 2013; Rubio and Nagy, 2015). This enables synchronous oscillations that temporally correlate firing patterns in otherwise heterogeneous networks (Galarreta and Hestrin, 1999; Deans et al., 2001; Bennett and Zukin, 2004; Vervaeke et al., 2010; Haas et al., 2011; Postma et al., 2011). In many regions of the nervous system, synchronous rhythmic oscillations serve as a mechanism to entrain the activity of distributed neuronal networks, and promote processes related to memory formation and retrieval, sensory perception, motor control, attention and plasticity (Deans et al., 2001; Hormuzdi et al., 2004; Van Der Giessen et al., 2008; Pereda, 2014).

Neurons express connexin36 (Cx36) and form gap junctions, which are the molecular substrate of electrical synapses. Functional plasticity of neuronal gap junctions in goldfish Mauthner cells (Pereda et al., 1998, 2003, 2004; Flores et al., 2010), and in both teleost and rodent retina (Li et al., 2009; Cachope and Pereda, 2012; Kothmann et al., 2012) involves interaction of Cx36 with the calcium/calmodulin-dependent kinase II (CaMKII). In this process, CaMKII binds Cx36 in a manner analogous to binding of this kinase to the NR2B subunit of the NMDA receptor and phosphorylates the connexin protein both *in situ* and *in vitro* (Alev et al., 2008). Blocking of Cx36–CaMKII binding and phosphorylation by decoy inhibition, and deletion of CaMKII binding sites, as well as inhibition of phosphorylation sites through site-directed mutagenesis and peptide inhibition, leads to a significant loss of the “run up” activity, the term coined by us to describe the plasticity of the process (Zoidl et al., 2002; Del Corsso et al., 2012).

Interaction of Cx36 with CaMKII endows electrical synapses with functional plasticity but is in competition with binding of calmodulin (CaM), another multifunctional Ca^2+^ signaling protein (Burgoyne, 2007), to a region overlapping with the CaMKII binding motif (Burr et al., 2005). At chemical synapses both CaM and the NMDA receptor subunit NR2B synergize to lock CaMKII in an active conformation conferring calcium-dependent plasticity (Bayer et al., 2001; Lisman et al., 2002; Bhattacharyya et al., 2016). A similar mechanism involving Cx36, CaM and CaMKII appears unlikely due to the overlapping binding site. Addressing this conflict, we have proposed a model predicting sequential, competitive processing of interaction between CaM, CaMKII, and Cx36 (Alev et al., 2008). To resolve this conflict, the neuroblastoma cell line Neuro2a was used to interrogate how calcium signaling converges on Cx36 through CaM binding. Using Förster resonance energy transfer analysis (FRET), we demonstrate that formation of the Cx36–CaM binding complex is dependent on the capacity of CaM to bind calcium and strengthened when intracellular calcium concentrations rise. The formation of the Cx36–CaM complex precedes arrival of the Cx36 protein at the gap junction plaque (GJP) or is established after removal of Cx36 from the GJP. Functional dye transfer/dye recovery assays in cell pairs under physiological conditions showed that Cx36–CaM binding endows GJPs with the capacity to exchange a fluorescent dye between adjacent cells. Pharmacological blocking of CaM or mutation of the critical amino acid W277 within the core CaM binding motif of Cx36 significantly diminished this property. This observation led us to propose a revised model of calcium signaling where the localization of Cx36 in the cell affects the participants (CaM and/or CaMKII) and consequently, the outcomes arising from these interactions. This model is additionally supported by detailed structural and kinetic view of the Cx36–CaM interaction using a combination of the NMR and calorimetric approaches. In summary, this study demonstrates that priming of Cx36 by CaM represents a critical stage in the creation of plasticity at electrical synapses.

## Materials and Methods

### Plasmid Constructs and Site-Directed Mutagenesis

Expression vectors contained full-length rat Cx36 [NM_019281, amino acids (aa) 1–321], CaM (NM_031969, aa 1–149), and CaMKIIa (NM_012920.1, aa 1–478) in variants of pEGFP-N1 expression vectors (Clontech Laboratories Inc., Mountain View, CA, USA) (Zoidl et al., 2002; Alev et al., 2008). Full-length in-frame cloning of CaM, the calcium insensitive mutant CaM_E1234Q_ (Maune et al., 1992; VanScyoc et al., 2002), as well as genetically encoded organelle markers (pER-DsRed2, pGolgi/DsRed2, pMito-DsRed2) (Fliegel et al., 1989; Rizzuto et al., 1989; Watzele and Berger, 1990) was completed in two steps. The open reading frames were synthesized as gBlocks [Integrated DNA Technologies Inc. (IDT), Coralville, IA, USA] and cloned into the TA cloning vector pJet1.2 (Thermo Fisher Inc., Mississauga, ON, Canada). Then the coding regions were isolated by restriction digest and cloned in-frame into pECFP-N1 or the pDsRed2-monomer expression vector. Mutant Cx36 plasmids (G276A, W277A, R278A) were generated using the Q5^TM^Site-Directed Mutagenesis Kit according to the manufacturer’s protocol [New England Biolabs Inc. (NEB), Boston, MA, USA]. Oligonucleotides were designed using the NEBaseChanger tool (NEB) and synthesized by IDT. **Table 1** summarizes the oligonucleotides utilized in this study (mutations depicted in bold and underlined). All plasmid constructs used on this study were sequence verified (Eurofins MWG Operon LLC, Huntsville, AL, USA).

**Table 1:** Oligonucleotides for Cx36 mutagenesis.

Gene	Primer ID	Sequence (5′–3′)	Application
Cx36	G276A-FP	TAACCATCTG**GCA**TGGCGGAAGA	Mutagenesis
	G276A-RP	AGTTCAGCCAGATTGAGC	
	W277A-FP	CCATCTGGGA**GCG**CGGAAGATCAAAC	
	W277A-RP	TTAAGTTCAGCCAGATTGAG	
	R278A-FP	TCTGGGATGG**GCG**AAGATCAAACTGGC	
	R278A-RP	TGGTTAAGTTCAGCCAGATTG	

*Point mutations are indicated in bold letters and underlined.*

### Cell Culture, Transient Transfection, and Western Blot

Neuroblastoma 2a (Neuro2a) cells (Olmsted et al., 1970) were cultivated in DMEM with 2 mM glutamine, 1% non-essential amino acids (NEAA), 1% penicillin and streptomycin (PS), and 10% fetal bovine serum (FBS) at 37°C in a humidified atmosphere with 5% CO_2_. Cells were seeded in 24-well plates or glass-bottom dishes (MatTek Corporation, Ashland, MA, USA) and transfected with 200 ng (single transfection) or 400 ng (double transfection) endotoxin-free plasmid DNA, using the Effectene transfection protocol (Qiagen Inc., Valencia, CA, USA). For western blot, whole cell protein lysates were prepared 48 h after transfection. The 20 μg protein was separated by 10% SDS-PAGE, transferred to 0.2 μm Midi format nitrocellulose membrane and processed using the iBind^TM^ Western System (Bio-Rad Inc., Mississauga, ON, Canada). Primary antibodies were diluted 1:1,000 [mouse anti-GFP, Roche; rabbit anti-GFP (FL), Santa Cruz Biotechnologies, Dallas, TX, USA] and 1:20,000 (mouse anti-β-actin; Sigma-Aldrich Chemie GmbH, Munich, Germany). The secondary antibodies (LI-COR Biosciences, St. Lincoln, NE, USA) were diluted 1:20,000 (donkey anti-rabbit IRDye680LT) or 1:20,000 (goat anti-mouse IRDye800CW). Signals were detected using the Odyssey^®^ CLx Infrared Imaging System (LI-COR Biosciences).

### Confocal Microscopy

Transfected cells were fixed with 4% paraformaldehyde for 20 min at RT, washed with PBS, and mounted with ProLong^®^ Antifade Mountant (Thermo Fisher Inc., Mississauga, ON, Canada) for imaging. Samples were visualized using a Zeiss LSM 700 confocal microscope with a Plan-Apochromat 63x/1.4 Oil DIC M27 objective and the ZEN 2010 program to control all hardware parameters. Images were collected by line averaging (4×) at high resolution (2048 × 2048 pixels) using single planes or z-stacks. The GJ frequency was quantified as described previously (Qu et al., 2009), by counting the number of EGFP-expressing cell pairs against the number of cell pairs having GJPs in images from randomly selected, non-overlapping visual fields collected from experimental replicates (*n* > 3). The GJ area was quantified using ImageJ. Here, the ImageJ free hand tool was used to draw the contours of GJs between cell pairs, followed by quantification of the GJ area using the measure tool. Mander’s overlap coefficients were calculated using the co-localization analysis if the ZEN 2010 program (Manders et al., 1993). Images were exported and further processed using ImageJ or the Imaris Scientific 3D/4D Image Processing & Analysis Software (Bitplane USA, Concord, MA, USA). Images were combined using Adobe Photoshop CS6 for presentation.

### FRET Analysis

The acceptor bleach protocol was established by testing different combinations of fluorescently tagged Cx36 and CaM proteins using a Zeiss LSM 700 confocal microscope (data not shown). In the experiments shown, Neuro2a cells were transiently transfected with DsRed-monomer and CFP tagged expression vectors and processed as described above. Baseline readings were measured prior to the acceptor bleach protocol. Here, DsRed-tagged proteins were bleached using the 555 nm laser line (set to 100% intensity) and the intensity change of CFP tagged proteins was recorded using the 405 nm laser line. The experiment was completed when the bleaching of the acceptor channel reached 10% of the initial intensity. FRET efficiency was then calculated by using the FRET efficiency formula: FRET_eff_ = (*D*_post_ -*D*_pre_)/*D*_post_, where *D*_post_ is the average intensity after the bleach, and *D*_pre_ is the average intensity before the bleach (Okamoto et al., 2004) after subtracting the values of the background (noise). The FRET distance (FRET_d_) between the interacting proteins was calculated using the formula: *R* = *R*_o_ ((1/*E*) - 1)^1/6^, where *R*_o_ is the reference distance between the two fluorescent tags (DsRed and ECFP: 5.09) (Erickson et al., 2003), and E is the FRET efficiency. The mean values of the intensity change and distance were calculated and formulated into graphical representations. Error bars denote the standard error of the mean (SEM) of each protein–protein interaction.

### Ethidium Bromide Uptake and Recovery after Photobleaching Assay

This assay is a variation of the ethidium bromide uptake assay previously reported (Prochnow et al., 2009; Kurtenbach et al., 2013). Transfected Neuro2a cells grown for 48 h in 3.5 cm MatTek cell culture dishes were incubated with 10 μM of ethidium bromide in complete growth medium for 10 min at 37°C and 5% CO_2._ MatTek chambers were placed in a live-cell imaging chamber and imaged at 37°C using a Zeiss 700 confocal microscope. Cell pairs expressing Cx36–EGFP were selected and a time-lapse baseline image was recorded (defined as 100%). Next, one cell was bleached using the 555 nm laser line with the intensity set to 100% laser power. The number of iterations in the bleaching protocol was terminated when 40–50% reduction in fluorescence was achieved. In this process, a volume of 36.2 μm × 36.2 μm × 1.6 μm was bleached using a non-confocal imaging mode. The recovery of ethidium bromide fluorescence in the center plane of this volume after bleaching was measured over time in confocal imaging mode. In all experiments, three regions of interest (ROI: R0, R1, R2) were selected for analysis. R0 was placed outside the cell pair to determine background the fluorescence. ROIs were placed inside the bleached cell close (R1) and distant (R2) to the GJP. The fluorescence recovery in percent was calculated using the formula:

$$\text{Fluorescence}\;\text{recovery}\;(\%)\;\text{=}\frac{\left({\text{F}}_{\text{3}\;\text{min}\;\text{post}\;\text{bleach}}\;\text{-}\;{\text{F}}_{\text{bleach}}\right)}{\left({\text{F}}_{\text{3}\;\text{min}\;(\text{pre-bleach})}\;\text{-}\;{\text{F}}_{\text{pre}\;\text{-}\;\text{bleach}}\right)}$$

### Pharmacology

Prior to imaging transfected cells were treated with the Ca^2+^ chelator BAPTA-AM (Thermo Fisher) (Ueda and Shah, 1992), ionomycin (Sigma-Aldrich) (Liu and Hermann, 1978), carbenoxolone (CBX; Sigma-Aldrich) (Li et al., 2001) or by applying the CaM antagonist W-7 (Sigma-Aldrich) (Weiss et al., 1982). The final concentrations in complete growth medium were 24 μM of BAPTA-AM, 10 μM of W-7, 50 μM of CBX, for 10 min or 2 μM ionomycin for 5 min at 37°C and 5% CO_2_. The final solvent concentration of ethanol in ionomycin treated cells was 0.002%.

### Protein Expression and Purification

A pET15 based (EMD Millipore, Etobicoke, ON, Canada) plasmid for expression of hexahistidine-tagged human calmodulin (His_6_-CaM) was a gift from Prof. Mitsu Ikura (University of Toronto). From a 1 L fermentation in Luria broth, milligram quantities of >95% pure His_6_-CaM were obtained by a two-step Ni-NTA affinity chromatography (Qiagen Inc., Toronto, ON, Canada) and gel filtration chromatography (Sephadex-75 HiLoad 16/60 column, GE Life Sciences) procedure. Final buffer conditions were 5 mM Tris-HCl, 100 mM NaCl, 0.05% NaN_3_, pH 7.5. The CaM–Cx36 complex pursued for structural determination was expressed as a 169 aa fusion protein consisting of His_6_-tagged human CaM (5–150), a short linker (GASTAAGS), and residues 276–292 of rat Cx36. A gene fragment encoding this fusion protein was synthesized by DNA2.0 (Menlo Park, CA, USA) and supplied in a T5-based expression vector (pJ441-kan). ^15^N, ^13^C labeled His_6_-CaM–Cx36 was obtained from a 1 L fermentation in M9 media containing 1 g (99%, ^15^N) ammonium chloride, 3 g (99%, ^13^C-glucose) and 0.5 g (98%, ^13^C–^15^N) algal extract (Cambridge Isotopes, Tewksbury, MA, USA). Final buffer conditions for NMR spectroscopy were 1.5 mM protein in 5 mM Tris-HCl, 100 mM NaCl, 5 mM CaCl_2_, 0.05% NaN_3_, pH 7.5 with 10% (v/v) ^2^H_2_O.

### NMR Spectroscopy

Spectra were acquired at 298 K with a 700 MHz Bruker Avance spectrometer equipped with a TXI cold probe. Backbone and side chain chemical shift assignments were obtained using a suite of standard heteronuclear, triple resonance experiments (HNCACB, CBCACONH, HNCO, HNCACO, HNCA, CCH-TOCSY, HCCH-TOCSY) with non-uniform Poisson gap sparse sampling at 10–15%. Distance restraint data were obtained from ^15^N-edited and ^13^C-edited 3D-NOESY spectra (120 ms mixing time). All datasets were processed with NMRPipe (Delaglio et al., 1995) and analyzed with CcpNmr Analysis (Skinner et al., 2015).

### Structure Determination

CcpNmr Analysis was used to integrate NOEs and calculate distance restraints based on an *r*^-6^ function spanning 1.8–5.5 Å with a reference distance set at 3.2 Å. These restrains were used as input to CYANA (Buchner and Güntert, 2015) along with Ca^2+^ coordination distance restraints, hydrogen bonding distance restraints and torsion angle restraints predicted by the DANGLE method embedded in CCPN Analysis. Allowed backbone torsion angles and side chain rotamers were enforced by the ramaaco and rotameraco methods embedded in the CYANA suite. The final representative ensemble of 20 structures based on lowest CYANA target function was selected from an initial calculation of 400 structures. As this ensemble demonstrated good geometry and bonding, no additional refinement was pursued.

### Databases

The structural ensemble was deposited in the Protein Data Bank under the accession number 2N6A.

### Isothermal Titration Calorimetry (ITC)

Isothermal titration calorimetry (ITC) measurements were performed on MicroCal VP-ITC calorimeter (MicroCal Inc., Northampton, MA, USA). The recombinant H_6_-CaM (affinity purified as described above) was titrated with Cx36 derived peptides reflecting the wild type (GSGWRKIKLAVRGAQAKRKSVYEIR; CanPeptide Inc., Montréal, QC, Canada) or the W227A mutant (GSGARKIKLAVRGAQAKRKSVY; BioBasic, Markham, ON, Canada). An optimal titration was achieved with 25 μM CaM in the reaction cell and 250 μM Cx36 peptide in the syringe, each in a buffer containing 50 mM NaCl, 5 mM BisTris pH 7.0, 5 mM CaCl_2_. After an initial injection of 2 μL, the bulk of the titration consisted of 34 successive 8 μL injections with an equilibration delay of 300 s. Heats of dilution were determined by titrating the same peptide solutions into buffer alone. Titration profiles corrected for the heat of dilution were fitted into one-binding-site model using MicroCal Origin v5.0. All values reported were calculated based on three individual experiments.

### Statistics

Statistical analysis and data presentation was performed using the IBM-SPSS and R-software packages. Results shown derive from experimental replicates with *n* ≥ 3. All data were analyzed for data distribution and subjected to Mann–Whitney *U* tests for independent samples or a paired *t*-test, when appropriate.

## Results

### Cx36–CaM at Gap Junctions in Neuro2A Cells

**FIGURE 1:**
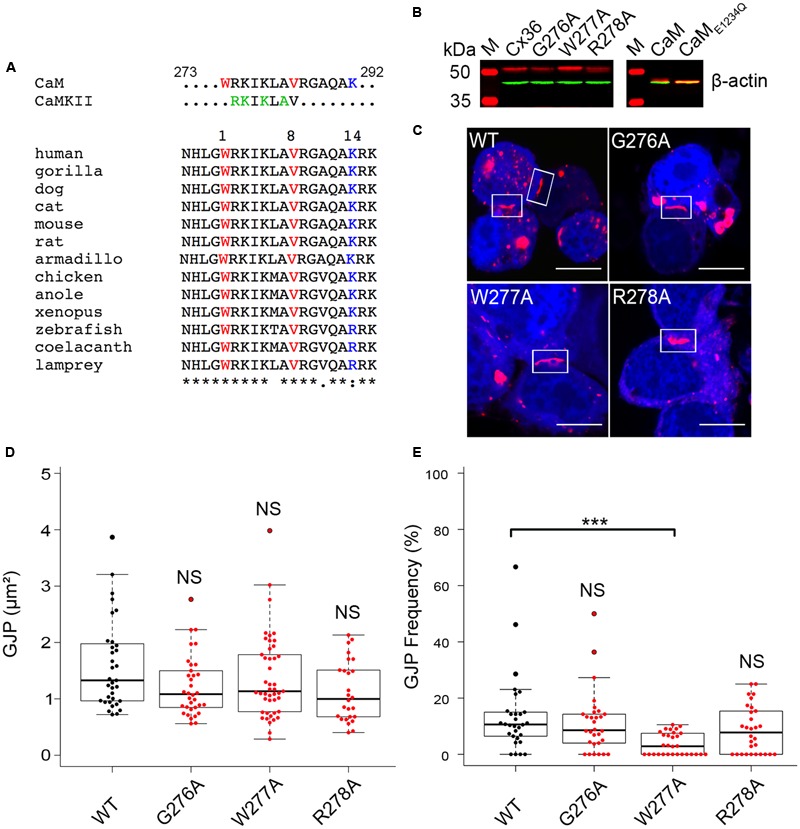
**Expression, localization, and gap junction plaque (GJP) formation of Cx36 in Neuro2a cells. (A)** Sequence alignment of Cx36 orthologs from major vertebrate phyla showing a conserved CaM-binding domain overlapping with the CaMKII pseudosubstrate binding site. The 1–8–14 positions of the CaM-binding motif are highlighted. **(B)** Western blot analysis of transiently transfected Neuro2a cells expressing Cx36 wild-type, Cx36 mutants, CaM, and CaM_E1234Q_. Expressed proteins were detected using an anti-EGFP antibody (in red), and an anti-beta-actin antibody (in green) as the control. M indicated the protein standard. **(C)** Rectangles indicate representative GJPs in Neuro2a cells double transfected with CaM (blue), Cx36 (red) wild-type, and mutant Cx36 proteins. Scale bar: 10 μm. **(D,E)** Quantification of gap junction size and frequency demonstrating that gap junction size is not affected by mutations of the core CaM binding region, but mutation W277A affects GJP frequency. Error bars box plot: maximum and minimum range with median; Mann–Whitney *U* test, ^∗∗∗^*p* < 0.001.

Previous work (Burr et al., 2005) and the sequence alignment of Cx36 orthologs representing major vertebrate phyla ranging from primitive fish to humans suggested the existence of a highly conserved potential CaM binding motif in the carboxy-terminus of Cx36 resembling a variation of the 1–8–14 subclass. This motif overlapped with a binding site for the calcium-dependent calmodulin kinase II (**Figure 1A**) (Alev et al., 2008).

The role of W277 in position 1 of the putative CaM-binding site was investigated after sequential alanine-scanning mutagenesis of positions 276 to 278 of the rat Cx36 protein. Western blot analysis confirmed exogenous expression of Cx36 wild-type and Cx36 mutants (G276A, W277A, and R278A) in single transfected Neuro2a cells 48 h post transfection (**Figure 1B**). A similar result was obtained with CaM and the Ca^2+^ insensitive mutant of CaM (CaM_E1234Q_; Maune et al., 1992) generated for this study (**Figure 1B**). The localization of Cx36, Cx36 mutants, and CaM was investigated in double-transfected cells 48 h post transfection. The formation of GJPs was observed in cells co-expressing CaM with wild-type Cx36 and mutant proteins (**Figure 1C**). A quantification of the GJP size revealed no significant differences in the GJP sizes by the mutations introduced (**Figure 1D**) (values in μm^2^; WT: 1.72 ± 0.20, G276A: 1.22 ± 0.09, *p* = 0.05, W277A: 1.37 ± 0.11, *p* = 0.19, R278A: 1.13 ± 0.11, *p* = 0.01). The number of cells with GJP formation in transfected cells was significantly reduced for mutant W277A, but not when WT was compared to G276A and R278A (**Figure 1E**) (values in %; WT: 13.68 ± 2.52, G276A: 11.24 ± 2.03, *p* = 0.5, W277A: 3.63 ± 0.72, *p* = 1.9 × 10^-5^, R278A: 8.69 ± 1.55, *p* = 0.12).

### Mutation W277A Alters Subcellular Distribution and Exchange between Cell Pairs

**FIGURE 2:**
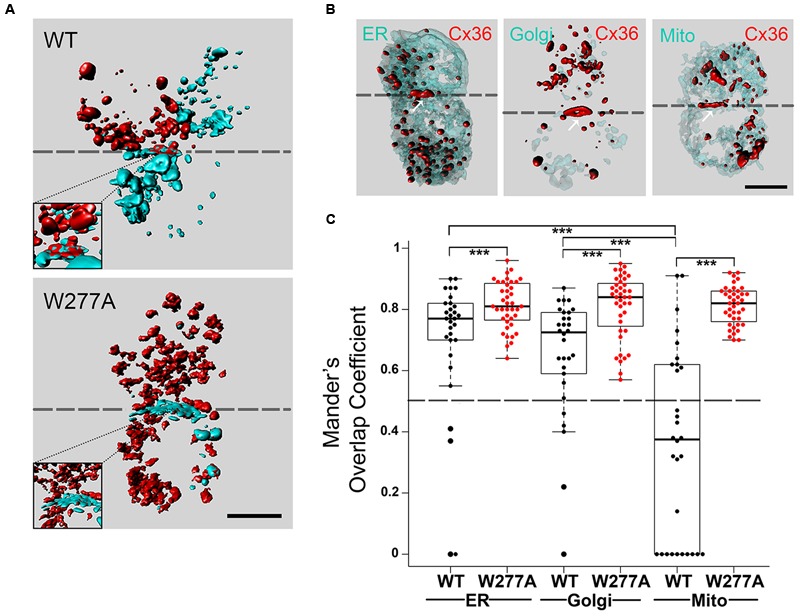
**Colocalization of Cx36 with organelle markers indicating interaction occurring as early as at the endoplasmic reticulum. (A)** Representative 3D images of Neuro2A cells expressing wild-type Cx36 or the W277A mutant. Note that cell pairs derived from single independent transfections tagging the proteins of interest with EGFP or DsRed monomer. The increased presentation of mixed red and turquoise vesicles in W277A transfected cell pairs suggests reduced GJP stability and increased protein turnover. Insets represent magnifications of the GJP region. **(B)** Neuro2a cells expressing wild-type Cx36–ECFP (in red) and DsRed monomer tagged organelle markers (endoplasmic reticulum, Golgi apparatus, and mitochondria, in light turquoise). The localization of Cx36 plaques is indicated with white arrows. **(C)** Mander’s overlap coefficients calculated with the ZEN 2010 program for Cx36 WT (*n* = 30) and W277A (*n* = 40) with the different organelle markers are shown with error bars indicating the maximum and minimum range. 3D images in **(A,B)** were created with Bitplane Imaris 7.6.4. Scale bars in **(A,B)**: 10 μm. Error bars box plot: maximum and minimum range with median; Mann–Whitney *U* test, ^∗∗∗^*p* < 0.01.

Two types of experiments were performed to clarify the distribution of Cx36 and W277A in Neuro2A cells. When single transfected Neuro2A cells expressing Cx36–EGFP or Cx36–DsRed monomer were paired and analyzed 48 h after transfection, 3D imaging of cell pairs showed mixed GJPs (see inset in **Figure 2A**; **Supplementary Figure S1**), and limited presentation of Cx36 in mixed vesicles. Cell pairs expressing the W277A mutation showed mixed GJPs (see **Supplementary Figure S2**), and an extensive display of vesicular structures exchanged between cell pairs, suggesting that the mutation altered GJP stability (**Figure 2A**).

Next, the subcellular localization of Cx36 was visualized using Neuro2a cells double transfected with EGFP tagged Cx36 and genetically encoded cell organelle markers for endoplasmic reticulum (ER), Golgi, and mitochondria (**Figure 2B**, **Supplementary Figure S3**). Wild-type Cx36 was predominantly localized in vesicles and at GJPs. Outside of GJPs, both 2D images (data not shown) and 3D reconstructions of images from double transfected cells showed Cx36 in vesicles of variable size embedded in, or attached to ER and Golgi (**Supplementary Figure S4**). Co-localization analysis revealed a significant overlap of Cx36 with ER (0.695 ± 0.0413, *n* = 30, *p* = 9.837 × 10^-6^) and Golgi organelles (0.651 ± 0.0355, *n* = 30, *p* = 4.209 × 10^-5^). The Mander’s overlap coefficient for Cx36 and mitochondria was significantly lower (0.356 ± 0.0413, *n* = 30) demonstrating that exogenously expressed, tagged wild-type Cx36 is not redistributed into this organelle type (**Figure 2C**; **Supplementary Figure S5**). The distribution of Cx36 in cell pairs, together with the localization of tagged Cx36 in ER, Golgi and GJPs is consistent with the subcellular distribution of other connexin proteins. When Cx36–W277A was expressed, the Mander’s overlap coefficient was significantly higher than wild-type Cx36 in all organelles (ER: 0.814 ± 0.012, *p* = 0.009; Golgi: 0.811 ± 0.017, *p* = 1.6 × 10^-5^; mitochondria: 0.812 ± 0.010, *p* = 2.22 × 10^-9^). The distinct subcellular distribution of the W277A mutant suggests a mechanism that interferes with the transport, formation and/or stabilization of GJPs in transfected Neuro2a cells.

### Cx36–CaM Binding Is Strengthened by Elevated Intracellular Calcium at Intracellular Vesicles

**FIGURE 3:**
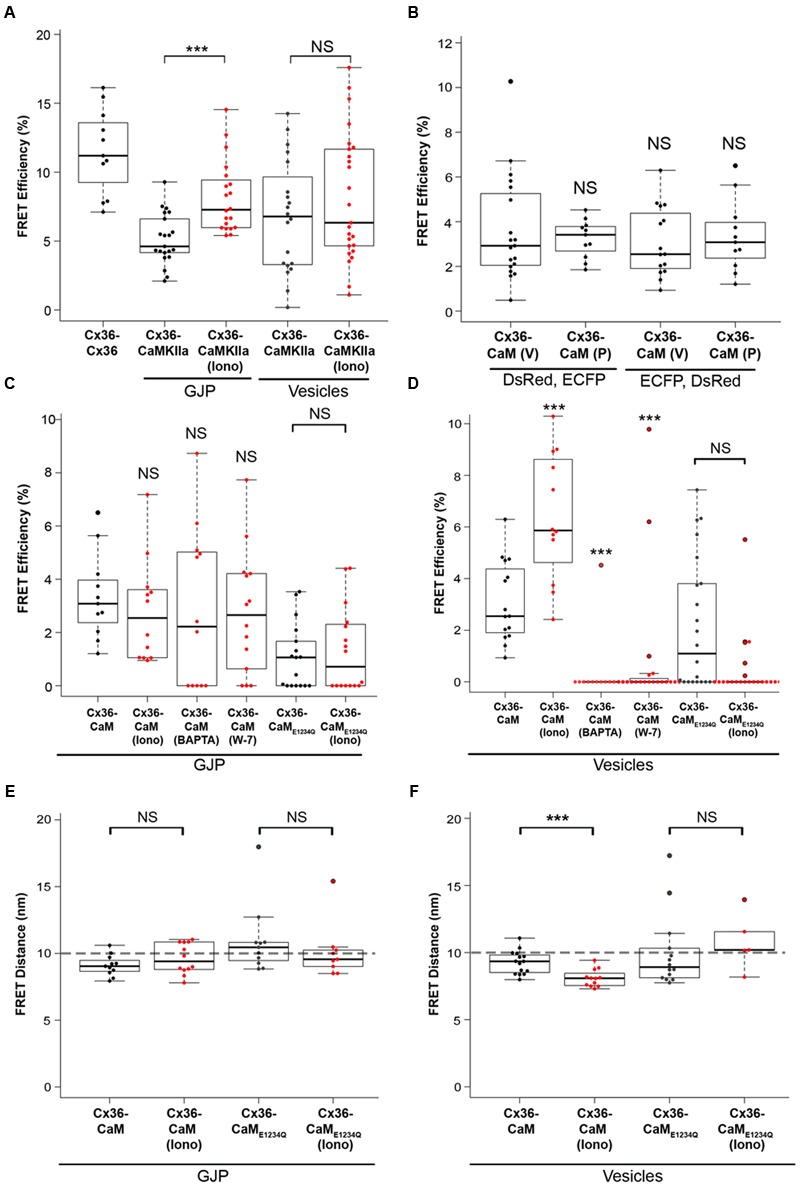
**FRET analysis of Cx36 and CaM interaction.** Neuro2a cells were transiently co-transfected, and FRET efficiencies (FRET_eff_) determined 48 h post transfection. **(A)** FRET_eff_ of Neuro2a cells expressing active FRET control pairs. **(B)** DsRed and ECFP protein tags are interchangeable and with minimal impact on FRET_eff_. **(C,D)** FRET_eff_ at GJPs and vesicles without and with 2 mM extracellular calcium [Ca^2+^]_E_ and 2 μM ionomycin in the presence of the pharmacological calmodulin blocker W-7 (10 μM), the calcium chelator BAPTA-AM (24 μM), or the Ca^2+^ insensitive CaM_E1234Q_ mutant. **(E,F)** FRET distance (FRET_dis_) highlighting that calcium-dependent binding of Cx36 and CaM is significantly shortened at vesicles. Abbreviations: Iono, treatment with 2 mM [Ca^2+^]_E_ and 2 μM ionomycin. V, vesicles; GJP, gap junction plaque. Error bars box plot: maximum and minimum range with median; Mann–Whitney *U* test significance, ^∗∗∗^*p* < 0.01, NS, not significant.

The interaction of Cx36–DsRed with CaM–ECFP in living cells was investigated using Fluorescence Resonance Energy Transfer (FRET) (**Figure 3**). The Cx36–ECFP and Cx36–DsRed assembly into mixed GJPs (**Figure 2A**) served as a positive FRET control. The FRET efficiency value (FRET_eff_ expressed in %) was 11.50 ± 0.93. The interaction of Cx36 with the Ca^2+^/calmodulin-dependent protein kinase II (CaMKII) has been shown previously (Alev et al., 2008; Del Corsso et al., 2012). Here, Cx36–ECFP and CaMKII–EYFP served as the second positive control demonstrating that intracellular calcium elevation by treatment with 2 mM extracellular calcium [Ca^2+^]_E_ and 2 μM ionomycin increased the FRET_eff_ significantly at GJPs (values in %: non-stimulated: 5.1 ± 0.4; stimulated: 8.1 ± 0.5, *p* = 0.0002), but not at vesicular structures (non-stimulated: 6.7 ± 0.9; stimulated: 8.1 ± 0.9, *p* = 0.33) (**Figure 3A**).

The FRET_eff_ for Cx36–DsRed and CaM–ECFP was comparable at GJPs and in cytoplasmic vesicles under physiological conditions without calcium stimulation (GJP: 3.23 ± 0.26; vesicles: 3.65 ± 0.54, *p* = 0.54) (**Figure 3B**). When the fluorescent protein tags (FRET pair I: Cx36–DsRed, CaM–ECFP; FRET pair II: Cx36–ECFP, CaM–DsRed) were switched (GJP: 3.35 ± 0.49, vesicles: 3.09 ± 0.40, *p* = 0.82) FRET_eff_ was not significantly different, suggesting that the protein tags had equal impact.

No significant changes to FRET_eff_ were observed at GJPs when intracellular calcium levels were raised (non-stimulated: 3.3 ± 0.5, stimulated: 2 mM [Ca^2+^]_E_ with 2 μM ionomycin; 2.8 ± 0.6, *p* = 0.33). Intracellular calcium reduction (24 μM BAPTA-AM; 2.8 ± 0.9, *p* = 0.42), or blocking of CaM with 10 μM (W-7; 2.7 ± 0.6, *p* = 0.44) had no impact on FRET_eff_. Co-transfection with the calcium insensitive CaM_E1234Q_ mutant (non-stimulated: 1.1 ± 0.3; stimulated: 1.3 ± 0.4, *p* = 0.85) reduced FRET_eff_ (**Figure 3C**). At vesicles, raising intracellular calcium ([Ca^2+^]_I_) levels significantly increased FRET_eff_ (non-stimulated: 3.10 ± 0.4, stimulated: 6.38 ± 0.71, *p* = 0.002), and led to a significant reduction after BAPTA-AM (0.30 ± 0.30, *p* = 0.9 × 10^-4^), or W-7 application (0.88 ± 0.56, *p* = 3.1 × 10^-5^) (**Figure 3D**). Similar to our measurements at the GJP, CaM_E1234Q_ at the vesicles showed a significantly reduced response upon [Ca^2+^]_I_ increase (non-stimulated: 2.18 ± 0.54, stimulated: 0.45 ± 0.27, *n* = 21, *p* = 0.005) (**Figure 3D**).

Next, FRET-Distance (FRET_dis_) for Cx36–CFP and CaM–DsRed was compared at GJPs and the vesicles. Typically, if two interacting proteins are within 10 nm distance, FRET can be achieved (threshold indicated by the dashed line). No significant difference at the GJPs (non-stimulated: 9.11 ± 0.24, stimulated: 9.61 ± 0.33, *p* = 0.33), but a significant reduction in FRET_dis_ was observed at cytoplasmic vesicles when [Ca^2+^]_I_ was increased, (non-stimulated: 9.27 ± 0.22, stimulated: 8.09 ± 0.19, *p* = 0.002). When CaM_E1234Q_ was used to test the interaction with Cx36 WT-CFP, neither at the GJP (non-stimulated: 10.93 ± 0.63, stimulated: 10.13 ± 0.53, *p* = 0.21) nor the vesicles (non-stimulated: 9.96 ± 0.58, stimulated: 10.81 ± 0.46, *p* = 0.23) was significantly different (**Figure 3E**). All values were below 10 nm except for CaM_E1234Q_, which indicated FRET was not achieved between wild-type Cx36 and CaM_E1234A_. We concluded that changes in [Ca^2+^]_I_ are relayed to Cx36 before reaching GJPs, and that the interaction between Cx36 and CaM is critically dependent on the capacity of CaM to bind calcium.

### W277 Is Critical for Calcium Dependent Cx36–CaM Binding

**FIGURE 4:**
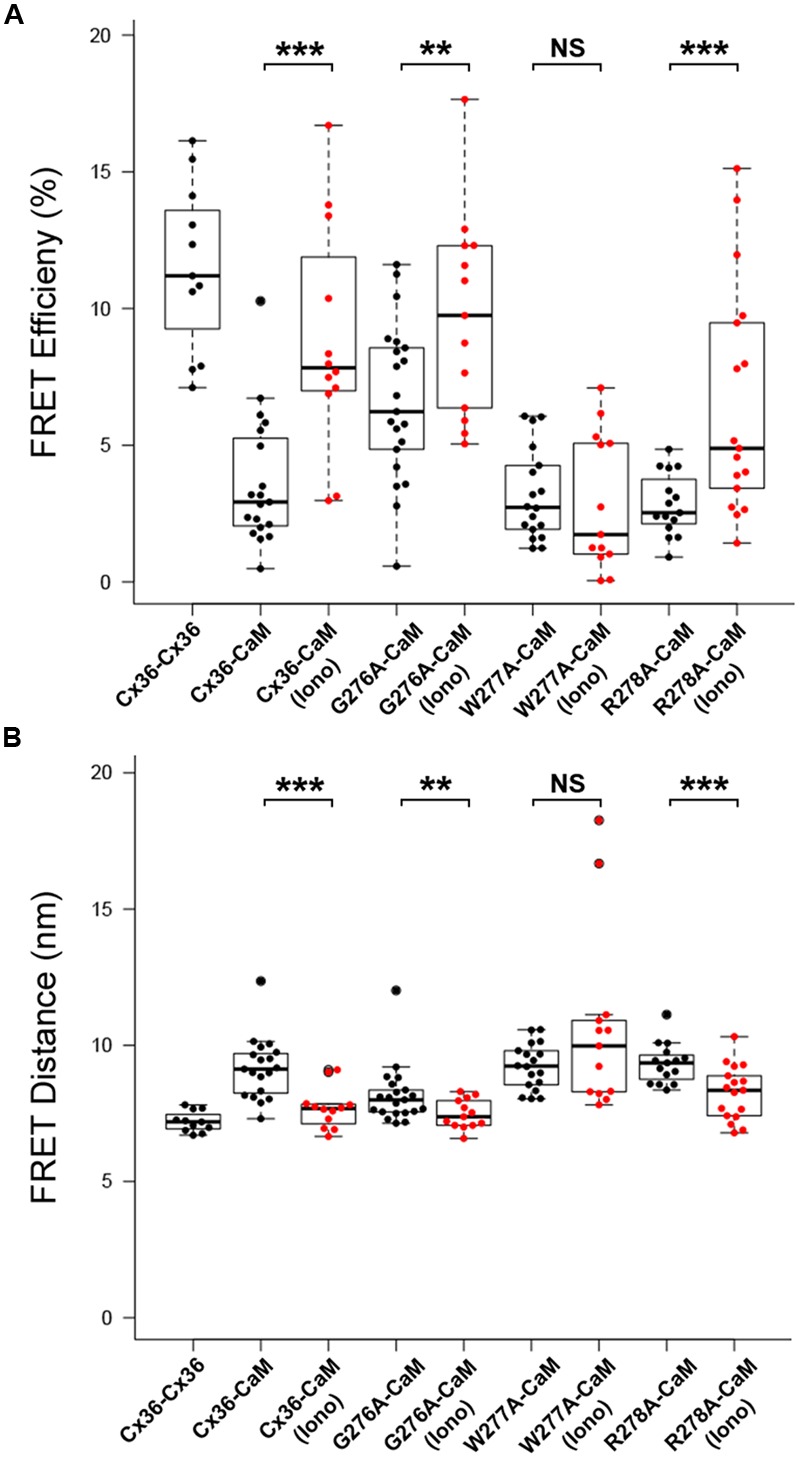
**Alanine scanning mutagenesis reveals W277 as the critical site for Cx36–CaM binding. (A)** FRET_eff_ at vesicles in Neuro2a cells. Cells expressed fluorescently tagged Cx36–ECFP and Cx36–DsRed monomer as the control. Wild-type Cx36–DsRed monomer and Cx36 mutants (G276A, W277A, R278A) in the presence of CaM were stimulated with 2 μM of ionomycin for 10 min. **(B)** FRET distance with the same parameters as FRET efficiency. The dashed line indicates the FRET distance threshold at 10 nm. Error bars box plot: maximum and minimum range with median; Mann–Whitney *U* test, ^∗∗^*p* < 0.05, ^∗∗∗^*p* < 0.01.

The role of the amino acid tryptophan in position 277 (W277) of Cx36 was investigated in calcium-dependent CaM binding together with the flanking amino acids 276 and 278. FRET_eff_ was determined for tagged Cx36 wild-type and Cx36 mutants in vesicles at physiological conditions, and after increasing [Ca^2+^]_I_ by stimulation with 2 mM [Ca^2+^]_E_ and 2 μM ionomycin. The FRET pair Cx36–ECFP and Cx36–DsRed served as the positive control (**Figure 4**). FRET_eff_ significantly increased in Neuro2a cells co-expressing CaM–DsRed and wild-type Cx36–ECFP (all values in %; non-stimulated: 3.65 ± 0.54, stimulated: 8.82 ± 1.19, *p* = 0.0003), the Cx36 mutants G276A (non-stimulated: 6.61 ± 0.63, stimulated: 9.74 ± 1.02, *p* = 0.02), or R278A (non-stimulated: 2.84 ± 0.29, *n* = 15; stimulated: 6.54 ± 1.02, *n* = 17, *p* = 0.003) (**Figure 4A**). In contrast, FRET_eff_ for W277A and CaM–DsRed were unaffected by the intracellular Ca^2+^ change (non-stimulated: 3.18 ± 0.39, *n* = 18; stimulated: 2.90 ± 0.69, *p* = 0.45).

This result was consistent with changes to the FRET distances (FRET_dis_) between tagged Cx36 and CaM after stimulation with 2 mM [Ca^2+^]_E_ and 2 μM ionomycin. Wild-type Cx36 (all values in nm; non-stimulated: 9.15 ± 0.26, stimulated: 7.69 ± 0.22, *p* = 0.0003), mutant G276A (non-stimulated: 8.16 ± 0.23, stimulated: 7.48 ± 0.15, *p* = 0.02), or mutant R278A (non-stimulated: 9.33 ± 0.19, stimulated: 8.26 ± 0.24, *p* = 0.003) showed a significant decrease in FRET_dis_, indicating that binding between both protein pairs increased (**Figure 4B**). In contrast, the FRET_dis_ for the Cx36 mutant W277A and CaM increased when intracellular Ca^2+^ was raised (non-stimulated: 9.22 ± 0.20, stimulated: 10.61 nm ± 0.91, *p* = 0.45). In summary, the FRET analysis suggested that tryptophan 277 is critical for CaM binding and that binding is sensitive to intracellular Ca^2+^ levels.

### Reduced Dye-Redistribution in Neuro2a Cells Expressing W277A

**FIGURE 5:**
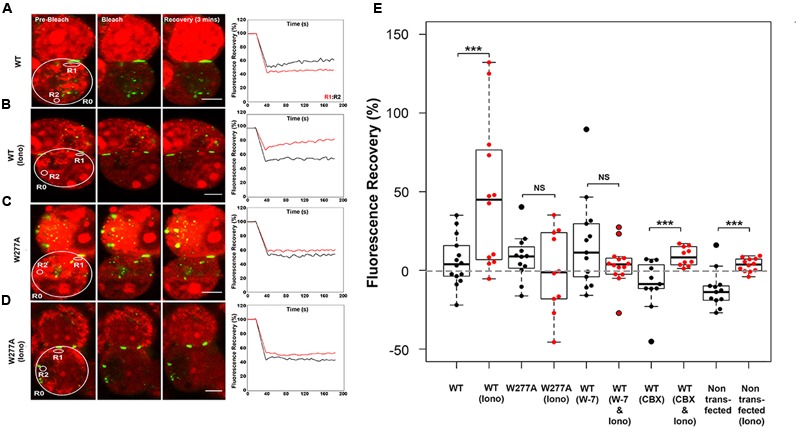
**Ethidium bromide dye uptake and redistribution after photobleaching. (A–D)** Cell pairs expressing Cx36 wild-type proteins or the W277A mutant. The left column highlights examples for each treatment condition, with the cells selected for photobleaching encircled (large circles) and cell positions of regions of interest indicated (small circles; R1, R2, and R0 = background). The right column shows traces representing fluorescence over time for regions of interests. Scale bars: 10 μm. **(E)** Quantification of fluorescence recovery in regions of interest. Treatments: Iono (2 mM [Ca^2+^]_E_ and 2 μM ionomycin), W-7 (10 μM W-7, calmodulin inhibitor), CBX (50 μM carbenoxolone, gap junction blocker). Error bars display the maximum and minimum range. NS, not significant. Paired *t*-test, *p*-values ^∗∗^ < 0.01, ^∗∗∗^ < 0.005.

The W277A mutation decreased the GJP frequency, but not the GJP size (**Figures 1D,E**), and caused a reduced response to [Ca^2+^]_I_ elevation (**Figures 4A,B**). To investigate impaired gap junction communication prompted by the W277A mutation, the non-invasive uptake and redistribution of ethidium bromide across GJPs under normal cell growth conditions was quantified using a method based on gap-FRAP (Abbaci et al., 2007). Pairs of Neuro2a cells expressing Cx36–ECFP or W277A–ECFP, and forming GJPs were selected for imaging (**Figures 5A–D**, left column). Sample traces for each experimental condition show the fluorescence in regions of interest (R1, red trace; R2, black trace) over the entire time course (**Figures 5A–D**, right column). Regions of interest were placed close to the GJP (R1) and at a more distant location (R2) in the same cell to capture recovery of ethidium bromide after photobleaching. Quantifications shown below (**Figure 5E**) demonstrated that treatment with 2 mM [Ca^2+^]_E_ and 2 μM ionomycin caused a significant ethidium bromide fluorescence recovery in Neuro2a cells expressing the Cx36–ECFP protein (values in %: non-stimulated: 5.82 ± 4.22, stimulated: 47.54 ± 13.56, *p* = 0.0045). Blocking of CaM with W-7 led to a significant reduction of fluorescence recovery when intracellular calcium levels were increased suggesting that CaM binding is critical for this response (non-stimulated: 16.60 ± 8.01, stimulated: 3.77 ± 3.41, *p* = 0.1427). Treatment with the gap junction blocker carbenoxolone (CBX) modulated fluorescence recovery (non-stimulated: -8.66 ± 4.65, stimulated: 8.91 ± 1.89, *p* = 0.0032). When Neuro2a cells expressed Cx36–W277A, no significant recovery was seen after the same treatment (non-stimulated: 8.12 ± 4.21, stimulated: -0.55 ± 8.35, *p* = 0.425). It was concluded that W277A expression impairs dye redistribution between cell pairs in a way similar to a gap junction blocker. Control experiments using non-transfected Neuro2a cells with and without ionomycin treatment showed limited recovery (Neuro2a without ionomycin treatment: -15.72 ± 17.52, Neuro2a with ionomycin treatment: 3.40 ± 1.23, *p* = 0.001). The recovery observed after CBX or ionomycin treatment likely reflects alterations to the biophysical properties of the cell membranes caused by the compounds applied.

### A High Resolution Solution Structure of Cx36 and CaM

**FIGURE 6:**
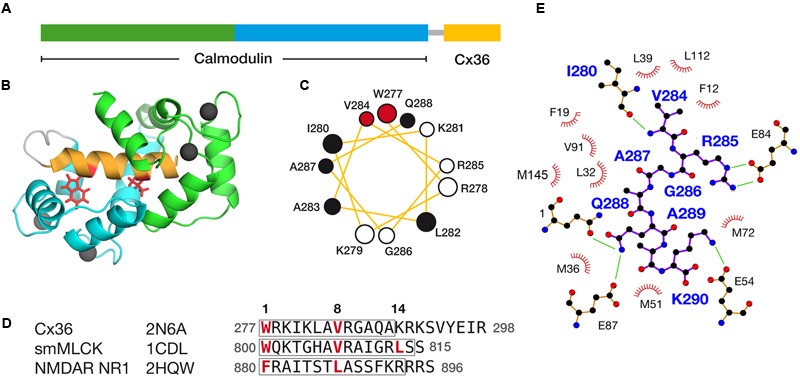
**Structural features of the Cx36–CaM complex. (A)** A schematic representation of the hybrid Cx36–CaM protein used for the NMR structural determination. The N- and C-terminal lobes are colored for reference, followed by a short linker leading into a region in the carboxy-terminal cytoplasmic tail of Cx36. **(B)** Cartoon representation of the lowest energy model in the ensemble of twenty solutions. Four calcium ions are depicted as balls and other features colored consistently with previous panels. **(C)** Helical wheel representation of Cx36 bound to CaM as determined by experimental observations. W277 and V284 at anchor positions 1 and 8 are colored red and other hydrophobic amino acids colored black. **(D)** Sequence alignment of Cx36, smooth muscle myosin light chain kinase (smMLCK), and NMDA receptor NR1 (NMDAR NR1). Key contact sites between CaM and these ligands are made at positions 1, 8, and 14. Boxes indicate the helical amino acids in each ligand. The ligand may extend amino-terminally before anchor position 1 but for clarity, only amino acids from position 1 are shown. **(E)** Ligand plot (Wallace et al., 1995) of key interactions observed in the Cx36–CaM complex. Green lines denote hydrogen bonds and ionic interactions.

Following a strategy used successfully in earlier structure determination (Ogura et al., 2012), a single-chain protein hybrid consisting of human CaM, a short glycine-rich linker, and the CaM binding region of rat Cx36 (**Figure 6A**) was expressed and uniformly ^13^C, ^15^N labeled for NMR spectroscopy. Amide ^1^H, ^15^N HSQC spectra of this CaM–Cx36 hybrid protein (**Supplementary Figure S6**) and a sample of ^15^N-labeled CaM in a 1:1 complex with a wild-type 25 amino acid long Cx36 carboxy-terminal tail peptide were similar suggesting that the structure of the CaM–Cx36 hybrid was an accurate representation of a binary Cx36–CaM interaction.

The Cx36–CaM hybrid protein structure was determined using 1100 distance restraints from ^15^N- and ^13^C-edited 3D-NOESY spectra, supplemented with a ^12^C-filtered, ^13^C-edited 2D-NOESY of a ^13^C-CaM/^12^C-Cx36 peptide complex (**Table 2**). The ensemble of 20 lowest energies structures is shown in **Supplementary Figure S7**. From the representative structure of the Cx36–CaM complex, CaM binds Cx36 in a well-known compact form (**Figure 6B**) with both Ca^2+^ saturated lobes enveloping a helical ligand with amphipathic features (**Figure 6C**). Based on the spacing of hydrophobic amino acids with W277 at anchor position 1, Cx36 resembles a 1–8–14 class ligand except that position 14 is occupied by K280 where a hydrophobic acid would typically be found. Accordingly, the Cx36–CaM protein structure compares favorably to other crystal structures of compact CaM complexes bearing 1–8–(14) ligands from smooth muscle myosin light chain kinase (Meador et al., 1992) (smMLCK; PDB:1CDL, 1.63 Å) and NMDA receptor NR1 C1 region (Ataman et al., 2007) (NMDAR NR1, PDB:2HQW, 1.81 Å) (**Figure 6D**). A contact map of Cx36–CaM from position 8 onward is shown in **Figure 6E**. The guanidino group of arginine at position 9 (R285 in Cx36) is balanced by ionic contacts from E84 in CaM. Likewise, the hydrophobic amino acid position 11 (A287 in Cx36) is supported by F19, L32, and V91 in CaM. At the penultimate contact position 14, the helix of Cx36 terminates earlier than smMLCK and NMDAR NR1 ligands. Despite this difference, three methionines in CaM (M36, M51, and M72) still contribute important hydrophobic contacts to Cx36. A lysine at position 14 in Cx36 is protruding from the CaM binding cleft toward a series of acidic amino acids in CaM (E47, D50, and E54). Taken together, a continuous network of contacts is observed between CaM and Cx36 thereby establishing Cx36 as bona fide ligand of CaM.

**Table 2:** Statistics for the ensemble of 20 structures of the CaM–Cx36 hybrid protein.

NOE restraints	
Total	1275
Intraresidue	432
Sequential	303
Medium range	269
Long range	271
Additional Restraints	
H-bond (H–O/N–O pairs)	58
Dihedral angle (ϕ/ψ pairs)	154
Ca^2+^ upper/lower restraint pairs	48
RMS violations	
NOE restraints (Å)	0.0223 ± 0.020
Dihedral angles (°)	0.77 ± 0.20
van der Waals clashes	5.9 ± 0.2
Ramachandran plot (%)	
Most favored regions	83.1
Additional regions	16.7
Generous regions	0.2
Disallowed regions	0.0
Structural precision RMSD	
Backbone atoms in ordered residues (Å)	0.63 ± 0.18
Heavy atoms in ordered residues (Å)	1.17 ± 0.14

*RMSDs for residues 11–18, 29–38, 46–55, 67–75, 82–92, 102–112, 118–126, 138–146, 156–168.*

**FIGURE 7:**
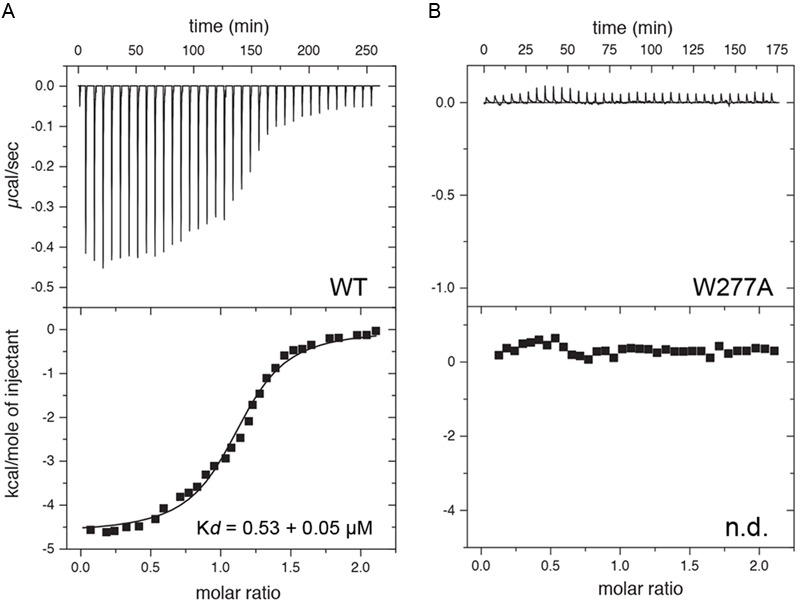
**Isothermal titration calorimetry (ITC) of Cx36 peptides and CaM.** Calorimetric traces and integrated isotherms acquired at 30°C for **(A)** Calcium saturated CaM titrated with a Cx36 derived peptide, and **(B)** Calcium saturated CaM titrated with the same peptide bearing a W277A substitution. No binding was detected between CaM and W277A mutant peptide. Abbreviations: *K*_d_, dissociation constant; n.d., not detected.

Finally, ITC was performed to determine the affinity of CaM for a 25 amino acid long peptide derived from the Cx36 cytoplasmic tail (**Figure 7**). As predicted from the NMR structure, the Cx36 peptide bound Ca^2+^-CaM stoichiometrically with a *K*_d_ of 0.53 ± 0.05 μM (*n* = 3). Highlighting the role of the amino acid W277 at the position 1 of the 1–8–(14) motif, no binding was observed for the peptide bearing a W277A substitution (*n* = 3). Consistent with the structure and biological assays described throughout this study, peptide binding was only observed with Ca^2+^ saturated CaM.

## Discussion

The interaction of the neuronal gap junction protein Cx36 with CaM was investigated in Neuro2a cells and the amino acid W277 identified as a critical residue in the CaM binding motif. The binding of Cx36 to CaM was demonstrated in intracellular vesicles and at GJPs, but only the interaction at vesicles showed a calcium-dependent increase, revealed by pharmacological blocking of CaM, manipulation of intracellular calcium or overexpression of a calcium insensitive CaM protein. Mutation W277A, as well as pharmacological blocking of CaM caused a significant reduction in dye transfer between cell pairs, implicating CaM binding to Cx36 in gap junction communication. *In vitro*, CaM binds Cx36 with a comparable affinity to other connexins with contacts that follow a 1–8–14 motif found in other CaM-binding proteins such as smMLCK, NMDAR-NR1, and MARCKS.

### The Cx36–CaM Binding Motif Is Distinct from Other Connexins

The novel details of CaM binding to rat Cx36 build on the original findings using mouse Cx36 and two perch Cx35 variants (Burr et al., 2005). The Cx36 binding motif is highly conserved across species from humans to lower vertebrates, like lamprey and zebrafish, and overlapping with a CaMKII binding site (Alev et al., 2008). In contrast, the CaM binding sites of Cx35/Cx36 are distinct from other connexins including Cx32, Cx43, Cx44, Cx46, and Cx50 in terms of ligand class (1–8 versus 1–5–10), location of the ligand binding sites, and resemble to date the only connexins activated by CaM (Zhou et al., 2007, 2009; Dodd et al., 2008; Myllykoski et al., 2009; Chen et al., 2011; Xu et al., 2012; Mruk et al., 2014).

The similarity of the Cx36 binding motifs across different species is reflected in comparable dissociation kinetics and thermodynamic characteristics found in mouse, the two perch Cx35 variants (Burr et al., 2005) and the rat Cx36 protein sequence investigated in this study. In more general terms, connexins bind CaM with dissociation kinetics and thermodynamic characteristics covering more than a 100-fold range (Dodd et al., 2008; Myllykoski et al., 2009; Zhou et al., 2009; Chen et al., 2011). Together, the specific localization of binding motifs, distinct subclasses and different dissociation kinetics suggest functional adaptations specific for each connexins protein.

The low affinity of Cx36 to CaM, at ∼500 nM, may have arisen due to the substitution of K290 at position 14 of typical 1–8–14 ligands like smMLCK, NMDAR-NR1, or MARCKS, which CaM encounters in the compact state. Furthermore, the less extensive array of hydrophobic contacts arising from the shorter side chains throughout Cx36, namely A283 and A287, cannot be compensated by larger hydrophobic amino at position 14 (K290 in Cx36). Regardless of the precise structural basis, a lower affinity may help Cx36 quickly sample CaM, CaMKII and possibly other proteins, depending upon the numerous signaling contexts it participates in throughout the life cycle of this protein.

### Calmodulin Confers Calcium Signaling to Cx36 Outside the GJP

Generally, CaM binding to connexins has been investigated in the context of gap junction communication, mainly focusing on implications for channel functions in the cell membrane. On the basis of the subcellular distribution of Cx36 and mutants thereof, we have expanded this focus, showing that binding already occurs before Cx36 reaches the cell membrane forming gap junctions, or after removal from the GJP. Interestingly, the W277A mutation caused retention in vesicles and showed a reduced gap junction coupling, reflecting in part similar observations made for Cx43 (Zhou et al., 2007). Further, gap junctions made by W277A are less frequent and more prone to removal as annular plaques found distributed in cell pairs, suggesting that decorating Cx36 with CaM is critical and most likely serving multiple purposes during the life cycle of this connexin.

### Ménage à Trios: Competition or Cooperation?

**FIGURE 8:**
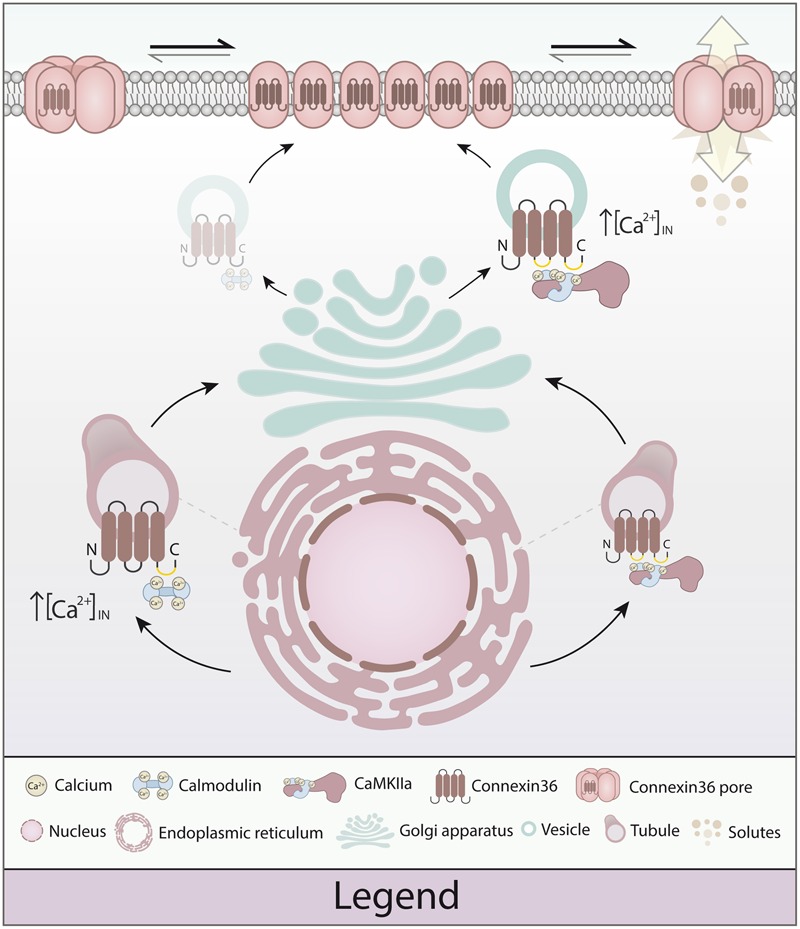
**Model of calmodulin (CaM), Ca^2+^/calmodulin dependent kinase 2a (CaMKIIa) and connexin 36 (Cx36) in plasticity of the electrical synapse.** Different to our original model, Cx36 can either bind calcium-activated CaM (right) or interact with CaMKIIa (left) at the vesicular level. The initial binding of CaM and Cx36 is critically dependent on elevated calcium concentrations. As the Cx36–CaM loaded vesicles traffic to the cellular membrane, the response to intracellular calcium activation shifts toward CaMKIIa interaction by calcium elevation. At the nexus, CaM serves as a kinase activator bound directly to CaMKIIa, and CaMKIIa plays an active part in modulating Cx36 nexus conductance (Illustration by Lesia Szyca, 2016).

We have previously proposed a model predicting the sequential, cooperative processing of interaction between CaM, CaMKII, and Cx36 at the GJP (Alev et al., 2008). The main results of this study are more consistent with a sequential and competitive process, sequestering calcium-dependent actions between vesicles and GJPs. To emphasize the new findings, we propose a revised model for plasticity at electrical synapses. As highlighted in **Figure 8**, both CaM and CaMKII have the capacity to bind Cx36 at vesicles, with CaMKII binding at physiological calcium levels, and CaM binding only when intracellular calcium is elevated. At GJPs, CaMKII is calcium-responsive, with CaM showing no further strengthening of binding in response to calcium increase. This sequence of actions differs significantly from the activation of NMDA receptors (Bayer et al., 2001), and lead us to conclude that electrical synapses share a portfolio of interacting proteins, but mechanisms of achieving plasticity do not appear to fully recapitulate those found at chemical synapses. Instead, CaMKII binding at Cx36 at GJP seems to be the critical interaction conferring calcium-dependent plasticity at electrical synapses, while calcium-dependent sensitization of CaM appears restricted to spatially separate compartments. This could endow electrical synapses with a tunable molecular machinery using calcium as the main regulator for activation through CaMKII, while interaction of Cx36 with CaM open new avenues for linking this channel to intracellular calcium signaling pathways.

What could be the function(s) of CaM binding to Cx36? The work by Burr et al. (2005) and our data suggest that CaM binding to Cx36 will occur when [Ca^2+^]_in_ levels are elevated. Such conditions typically are found during synaptic activity in neurons (Gamble and Koch, 1987). Since elevation of [Ca^2+^]_in_ increases Cx36–CaMKII, but not Cx36–CaM interaction at GJP, we conclude that calcium signals converge through CaM onto Cx36 before the protein reaches the GJP, ruling out a major role in plasticity.

It is reasonable to speculate that Cx36–CaM binding at vesicles may be sensitive to competition with other proteins with higher CaM affinity, suggesting that CaM binding could be orchestrated and/or sequestered by calcium affinities of CaM relative to CaMKII, or other, yet unidentified calcium binding proteins, or those activated or sensitized by calcium. Prior to its arrival at GJPs, Cx36–CaM could promote vesicular transport to the cell membrane and/or post-translational modifications affecting the oligomerization of connexins in the Golgi complex (Musil and Goodenough, 1993), as well as the assembly or disassembly of Cx36 into GJPs, potentially in a way similar to glutamate receptor trafficking to dendritic spines (Korkotian and Segal, 2007; Loo et al., 2014). Alternatively, calcium-dependent binding of CaM to Cx36 could link functional Cx36 channels in the ER directly to intracellular calcium signaling as suggested for Panx1, Panx3 or the transient receptor potential vanilloid 1 channel (TRPV1) (Vanden Abeele et al., 2006; Gallego-Sandin et al., 2009; Ishikawa et al., 2011), or by limiting calcium leakage from the ER as exemplified by CaM binding to Sec61 alpha (Erdmann et al., 2011). Addressing these consequences in future experiments will close this important knowledge gap.

## Author Contributions

RS, ES, CB, CZ, and LD performed experiments. All authors analyzed data and were involved in the manuscript preparation. DS and GZ designed the study and finalized the manuscript.

## Conflict of Interest Statement

The authors declare that the research was conducted in the absence of any commercial or financial relationships that could be construed as a potential conflict of interest.

## Supplementary Material

FIGURE S1 **Representative 3D images of Neuro2A cells expressing wild-type Cx36, tagged with EGFP, or DsRed monomer.** The original projection based on the raw Z-stack before processing is shown top left. By rotating the 3D image and zooming into structural details of the Cx36 distribution it is possible to highlight the exchange of Cx36 proteins between adjacent cells connected by a GJP. Note that GJPs share a mixture of EGFP (in turquoise) and DsRed monomer (in red) tagged Cx36 proteins, whereas redistribution between cells as indicated by red and turquoise vesicles is low (Scale bar: 10 μm).

FIGURE S2 **Representative 3D images of Neuro2A cells expressing the Cx36 W277A mutant.** The original projection based on the raw Z-stack before processing is shown top left. Note the mixed red and turquoise GJP and the increased redistribution of red and turquoise vesicles between adjacent cells indicating the reduced stability of the GJP and increase in protein turnover (Scale bar: 10 μm).

FIGURE S3 **Western blot detection of ER and Golgi tagged EGFP after transient expression in Neuro2a cells (in red, position indicated by lines).** β-actin expression was used as internal protein standard.

FIGURE S4 **Representative 2D co-localization images of Neuro2A cells expressing wild-type EGFP tagged Cx36 and genetically encoded cell organelle markers for endoplasmic reticulum (ER), Golgi, and mitochondria Cx36.** White areas indicate co-localization between Cx36 and organelle markers. (Scale bar: 10 μm).

FIGURE S5 **Representative 3D images of Neuro2a cells expressing wild-type Cx36–ECFP (in red) and DsRed monomer tagged mitochondria (in light turquoise).** This image is a typical example showing the lack of co-localization between Cx36 and mitochondria (Scale bar: 10 μm).

FIGURE S6 **A ^15^N-edited HSQC spectum of ^13^C, ^15^N-labeled CaM–Cx36 hybrid protein acquired at 700 MHz.** Cx36 specific amino acids are labeled red. Amino side chain resonances of asparagine and glutamine are indicated by dashed lines. An asterisk indicates an unassigned resonance. A plus sign indicates the position of a resonance below the contour limit of the plot.

FIGURE S7 **Superposition of the ensemble of 20 CaM–Cx36 NMR solution structures deposited in the Protein Data Bank (2N6A).** The protein is presented as a Cα ribbon (CaM lobe 1, green; CaM lobe 2, blue; linker, gray; Cx36, orange) and the calcium ions are presented as spheres.
