# Supplementary figures and images for: Structural and Functional Consequences of Connexin 36 (Cx36) Interaction with Calmodulin
Source: Front Mol Neurosci. 2016 Nov 18;9:120. doi: 10.3389/fnmol.2016.00120 (PMC5114276; doi:10.3389/fnmol.2016.00120)

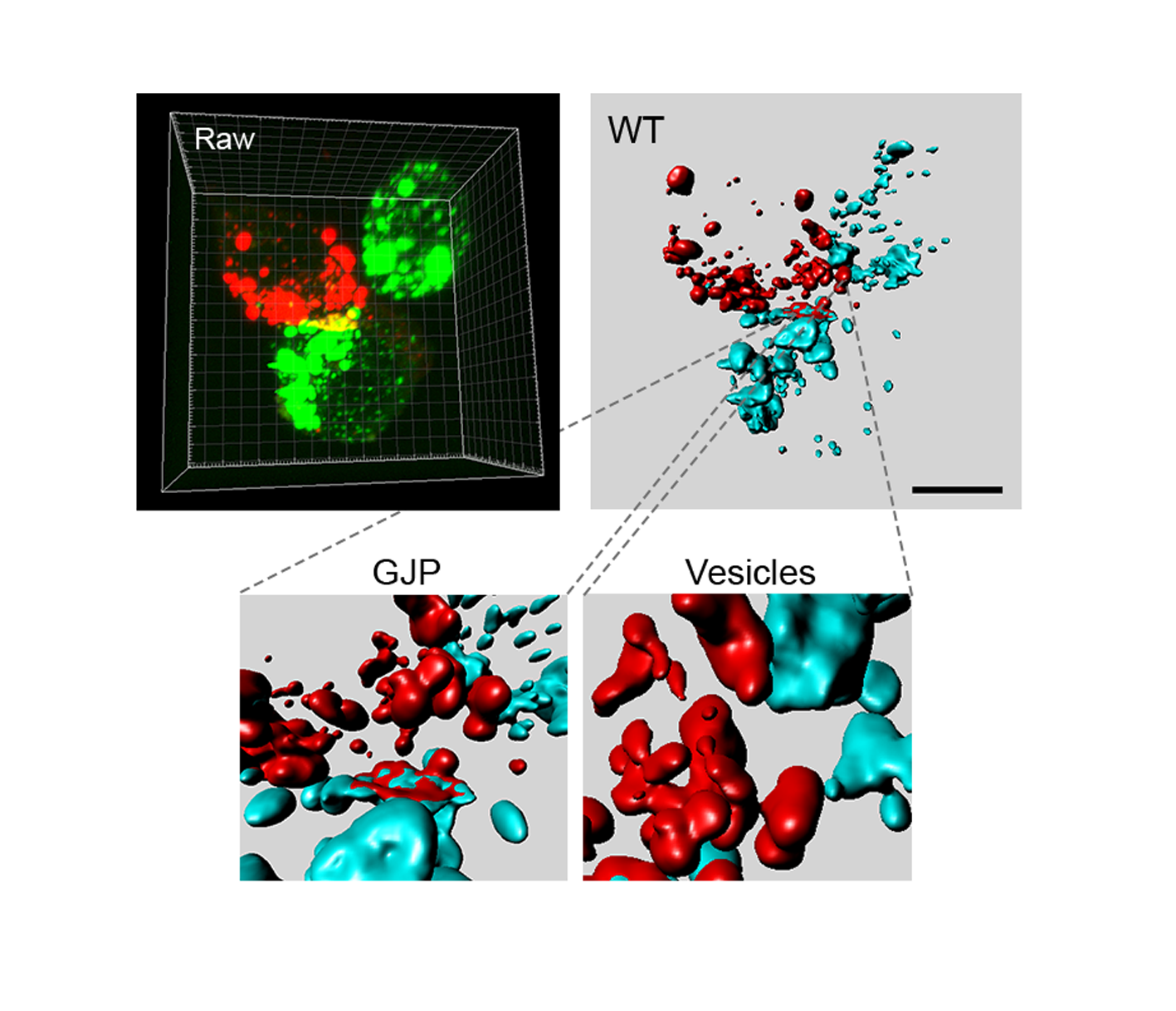

Supplement: FIGURE S1 — Representative 3D images of Neuro2A cells expressing wild-type Cx36, tagged with EGFP, or DsRed monomer. The original projection based on the raw Z-stack before processing is shown top left. By rotating the 3D image and zooming into structural details of the Cx36 distribution it is possible to highlight the exchange of Cx36 proteins between adjacent cells connected by a GJP. Note that GJPs share a mixture of EGFP (in turquoise) and DsRed monomer (in red) tagged Cx36 proteins, whereas redistribution between cells as indicated by red and turquoise vesicles is low (Scale bar: 10 μm). [file Image_1.TIF]

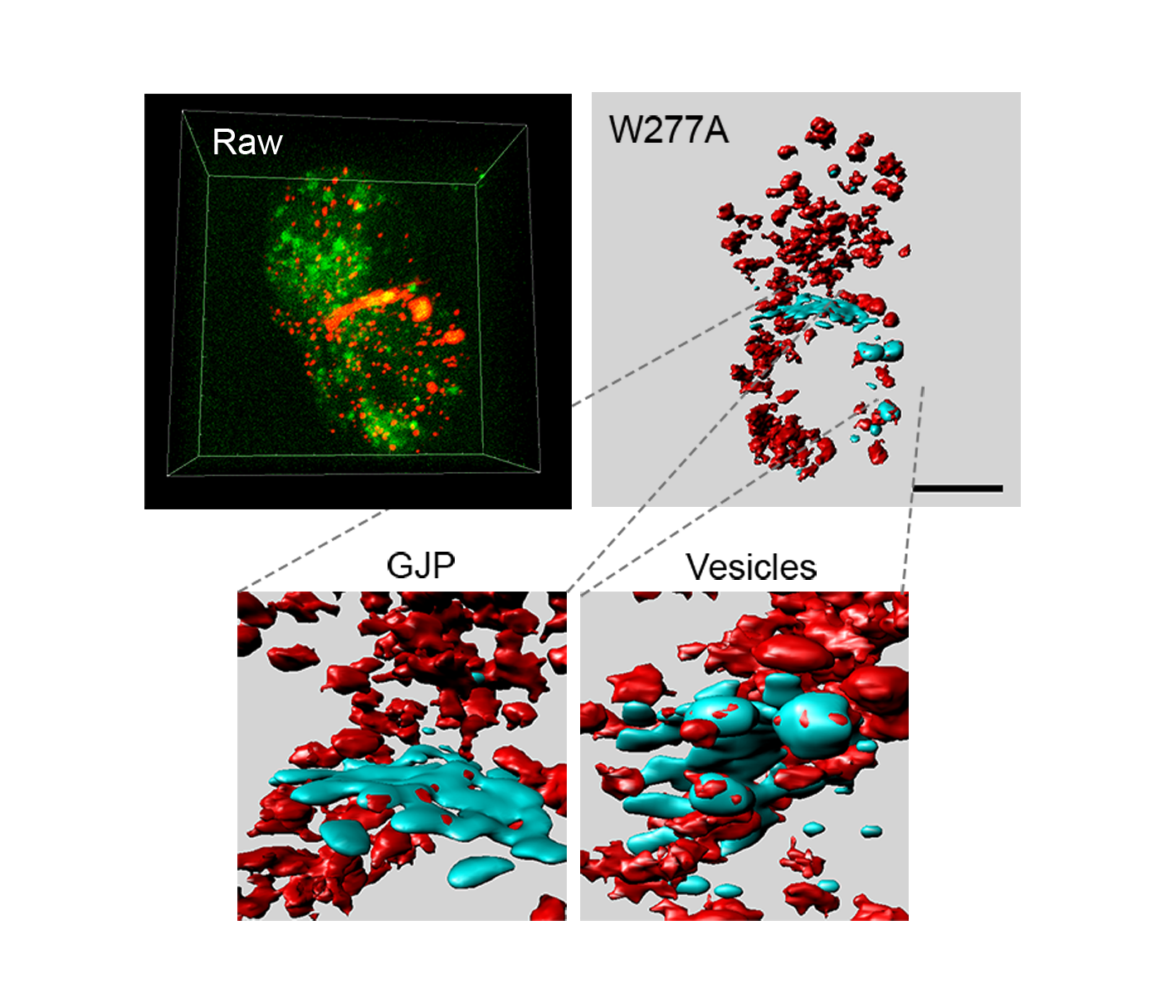

Supplement: FIGURE S2 — Representative 3D images of Neuro2A cells expressing the Cx36 W277A mutant. The original projection based on the raw Z-stack before processing is shown top left. Note the mixed red and turquoise GJP and the increased redistribution of red and turquoise vesicles between adjacent cells indicating the reduced stability of the GJP and increase in protein turnover (Scale bar: 10 μm). [file Image_2.TIF]

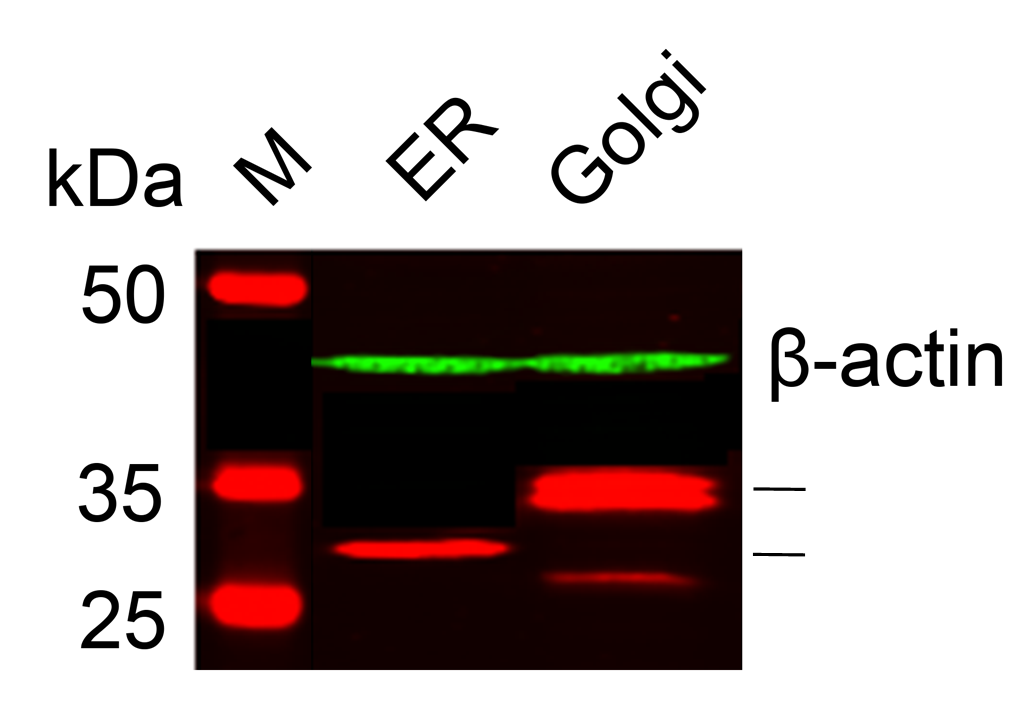

Supplement: FIGURE S3 — Western blot detection of ER and Golgi tagged EGFP after transient expression in Neuro2a cells (in red, position indicated by lines). β-actin expression was used as internal protein standard. [file Image_3.TIF]

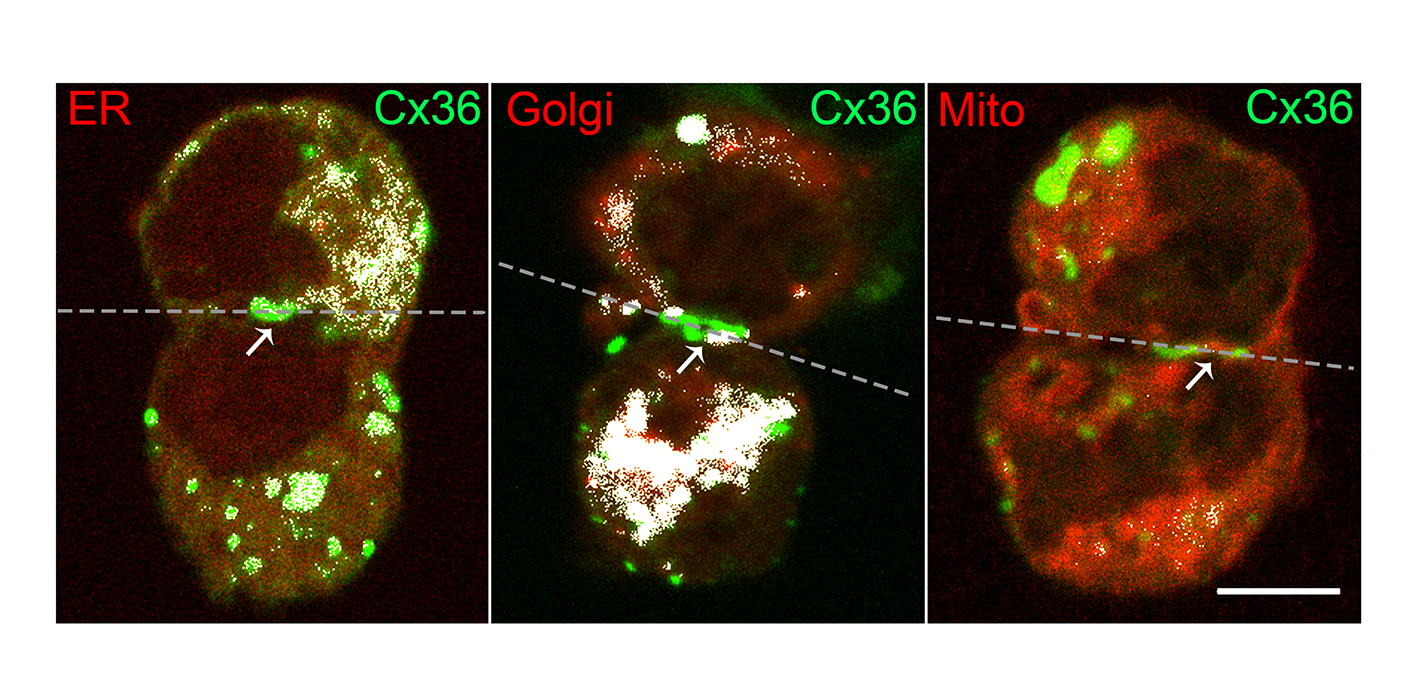

Supplement: FIGURE S4 — Representative 2D co-localization images of Neuro2A cells expressing wild-type EGFP tagged Cx36 and genetically encoded cell organelle markers for endoplasmic reticulum (ER), Golgi, and mitochondria Cx36. White areas indicate co-localization between Cx36 and organelle markers. (Scale bar: 10 μm). [file Image_4.TIF]

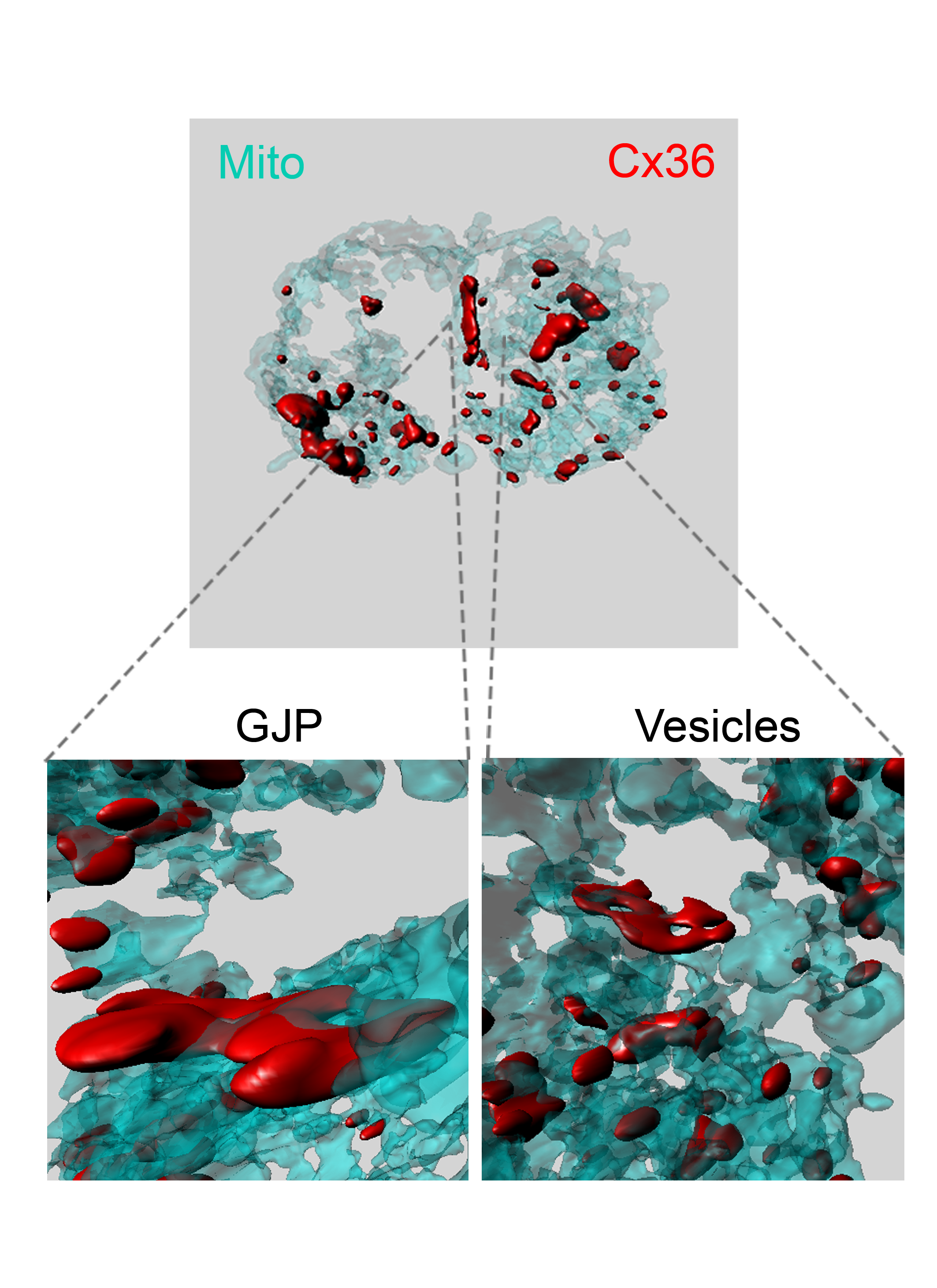

Supplement: FIGURE S5 — Representative 3D images of Neuro2a cells expressing wild-type Cx36–ECFP (in red) and DsRed monomer tagged mitochondria (in light turquoise). This image is a typical example showing the lack of co-localization between Cx36 and mitochondria (Scale bar: 10 μm). [file Image_5.TIF]

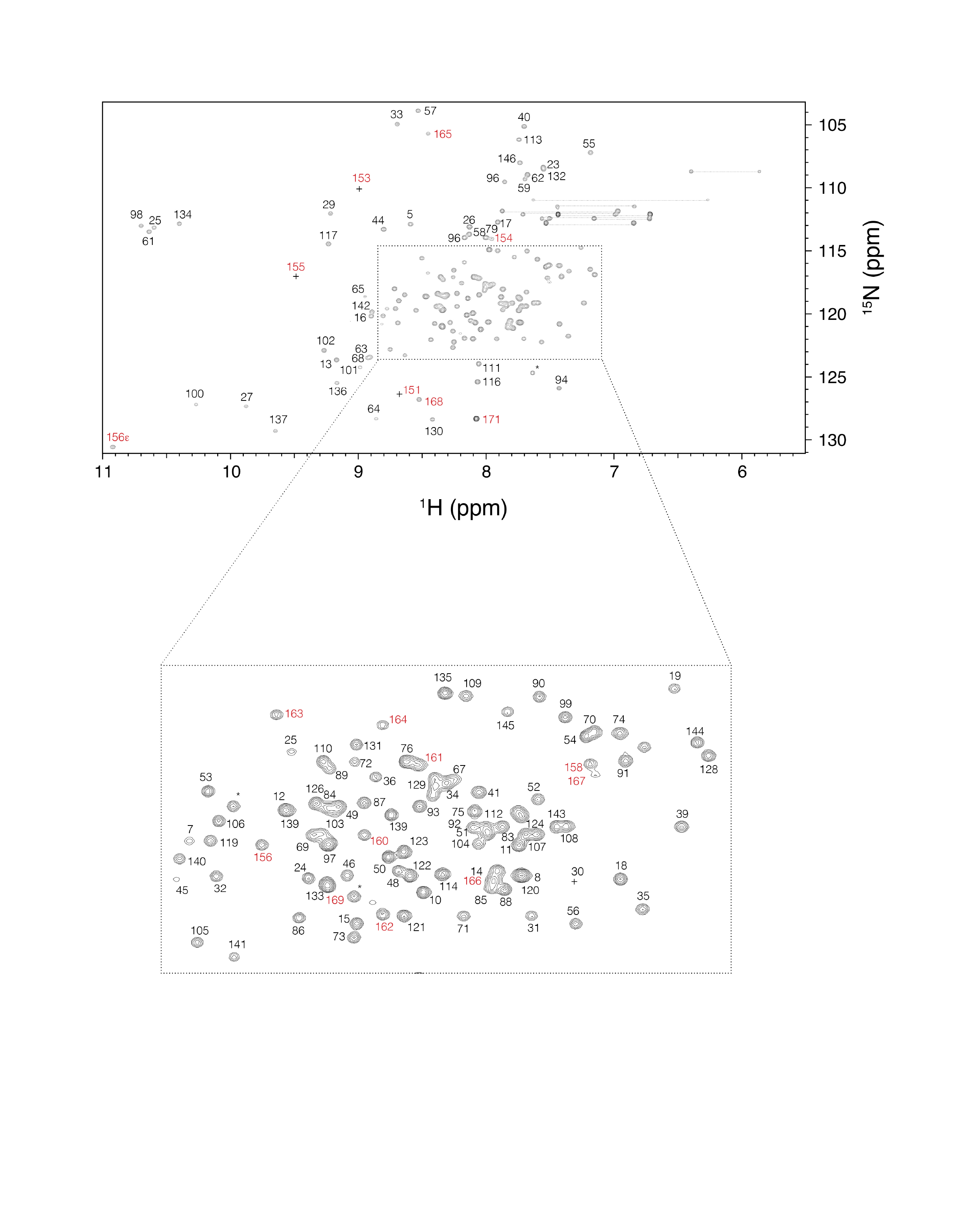

Supplement: FIGURE S6 — A 15N-edited HSQC spectum of 13C, 15N-labeled CaM–Cx36 hybrid protein acquired at 700 MHz. Cx36 specific amino acids are labeled red. Amino side chain resonances of asparagine and glutamine are indicated by dashed lines. An asterisk indicates an unassigned resonance. A plus sign indicates the position of a resonance below the contour limit of the plot. [file Image_6.TIF]

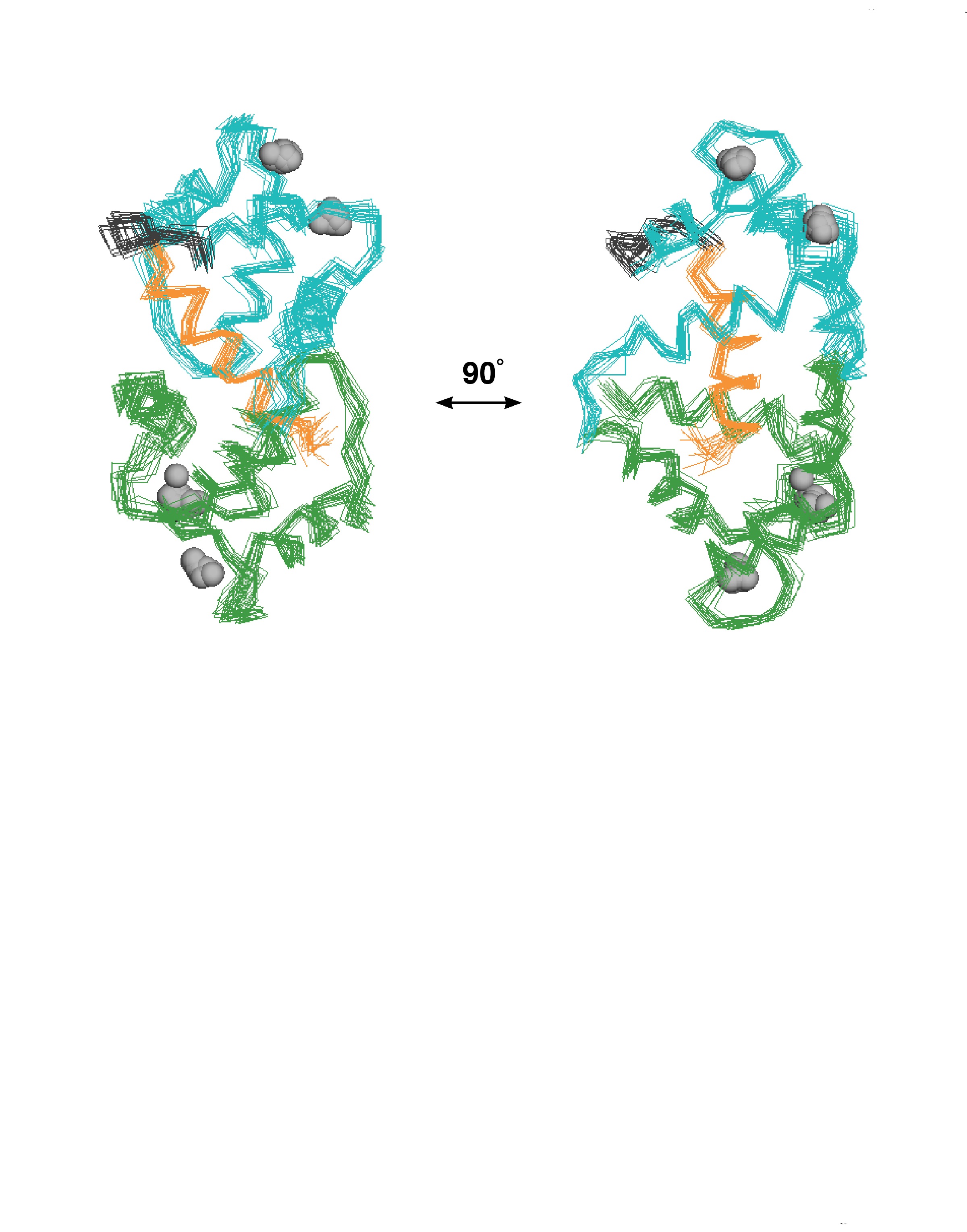

Supplement: FIGURE S7 — Superposition of the ensemble of 20 CaM–Cx36 NMR solution structures deposited in the Protein Data Bank (2N6A). The protein is presented as a Cα ribbon (CaM lobe 1, green; CaM lobe 2, blue; linker, gray; Cx36, orange) and the calcium ions are presented as spheres. [file Image_7.TIF]
